# Draft Genome Sequence of *Paenibacillus* sp. Strain OT2-17, a Triclosan-Degrading Rhizobacterium

**DOI:** 10.1128/MRA.01596-19

**Published:** 2020-04-16

**Authors:** Ashley M. Garcia, Priscilla P. Carlo, Monica O. Mendez

**Affiliations:** a Department of Biology & Chemistry, Texas A&M International University, Laredo, Texas, USA; University of Arizona

## Abstract

We report the draft genome sequence of a strain (OT2-17) of *Paenibacillus* isolated from the rhizosphere of onions irrigated with triclosan. Strain OT2-17 demonstrated the use of triclosan as the sole carbon source. A genome assembly of approximately 5.8 Mb was generated with a calculated G+C content of 45.5%.

## ANNOUNCEMENT

Triclosan, a broad-spectrum antimicrobial, accumulates in soils and crop tissues and induces antibiotic cross-resistance in clinical bacteria (1–3). Removal of triclosan to prevent impacts on agricultural systems would be favorable. As we report here, the biodegradation activities of *Paenibacillus* spp. make this an important resource for industrial and bioremediation applications (4, 5).

**FIG 1:**
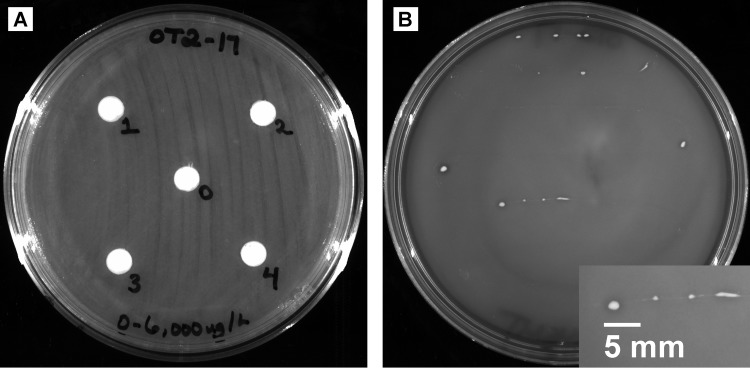
(A) *Paenibacillus* sp. strain OT2-17 demonstrated no sensitivity to triclosan at 0, 750, 1,500, 3,000, and 6,000 μg liter^−1^ (disks 0 to 4, respectively). (B) Triclosan degradation was indicated by the growth and presence of clearing around OT2-17 on a chloride-free minimal salt medium amended with triclosan (MSMT; 2 g liter^−1^) as the sole carbon source. The inset image shows an enlarged portion of the streak.

*Paenibacillus* sp. strain OT2-17 was isolated from the rhizosphere of onions irrigated with 0.15 μg liter^−1^ triclosan for 8 weeks (2). Rhizobacteria were collected from 1-cm sections of root tips and basal plates as previously described (6). The slurry was serially diluted in 1× phosphate-buffered saline (PBS) and plated onto R2A agar. After an initial 5-day incubation at 23°C, a small circular white colony was isolated. Triclosan resistance (>6,000 μg liter^−1^) was determined using the Kirby-Bauer disk diffusion assay (7) by inoculation onto Mueller-Hinton II agar and disks impregnated at 0, 750, 1,500, 3,000, and 6,000 μg liter^−1^ triclosan. No zones of inhibition (Fig. 1A) were observed after 48 hours (23°C). OT2-17 was then cultured on a chloride-free minimal salt medium (8) with triclosan (MSMT; 2 g liter^−1^) as the sole carbon source. Triclosan degradation was indicated by an intermediate level of growth (14-day incubation, 23°C) and clearing around the culture (Fig. 1B). Genomic DNA was purified from a 5-ml culture in R2B (24 hours, 23°C) using the Wizard genomic DNA purification kit (Promega, Madison, WI). Whole-genome sequencing was conducted at the Hubbard Center for Genome Studies (Durham, NH, USA) by preparing genome libraries using Nextera library preparation kits (Illumina) for the Illumina HiSeq 2500 platform. The 250-bp paired-end reads were paired and trimmed using the following parameters in Trimmomatic v.0.32: paired-end mode, SLIDINGWINDOW:4:15, and MINLEN:36 (9). Trimmed reads (74,150 reads) were assembled *de novo* using SPAdes v.3.13.0 with default settings (10). Contigs with <500 bp and those containing contaminants were removed. Genome assembly statistics were evaluated using QUAST v.5.0.2 (11), and average coverage (155×) was calculated by mapping FASTQ reads to the assembled contigs with the Burrows-Wheeler Aligner MEM (BWA-MEM) algorithm (12). Genome annotation was performed using the NCBI Prokaryotic Genome Annotation Pipeline (PGAP) v.4.10 (13).

The resulting genome assembly was 5,757,485 bp with 1,067 contigs (largest contig, 247,693 bp), an *N*_50_ value of 11,011 bp, and a G+C content of 45.5%. Totals of 5,823 genes, 5,747 coding sequences, 249 pseudogenes, 18 rRNAs, and 54 tRNAs were identified. BLASTn was used to identify isolate OT2-17 as Paenibacillus peoriae KCTC 3763T (99.66% identity) using the 16S rRNA sequence (14); the genome was most similar to P. polymyxa CR1 and *P. peoriae* HS311 (98.58% and 98.54% orthologous average nucleotide identity, respectively) according to the OAT tool (15). Recent genome comparisons of *Paenibacillus* spp. suggest that new classifications are needed (16); therefore, we have designated OT2-17 as a strain of *Paenibacillus*.

### Data availability.

This whole-genome shotgun project has been deposited in DDBJ/EMBL/GenBank under the accession no. WUDQ00000000. The version described in this paper is the first version, WUDQ01000000. The raw Illumina reads are available as BioProject PRJNA597179 in the Sequence Read Archive (SRA).
